# Insuficiência Cardíaca com Fração de Ejeção Intermediária - Condição Temporária ou um Grupo Específico?

**DOI:** 10.36660/abc.20210482

**Published:** 2021-08-09

**Affiliations:** 1 Universidade Federal do Ceará Faculdade de Medicina FortalezaCE Brasil Faculdade de Medicina da Universidade Federal do Ceará, Fortaleza, CE - Brasil.; 2 Centro de Arritmia do Ceará FortalezaCE Brasil Centro de Arritmia do Ceará, Fortaleza, CE - Brasil.; 3 Hospital de Messejana Dr. Carlos Alberto Studart Gomes FortalezaCE Brasil Hospital de Messejana Dr. Carlos Alberto Studart Gomes, Fortaleza, CE - Brasil.; 4 Universidade de Fortaleza Centro de Ciências da Saúde FortalezaCE Brasil Universidade de Fortaleza Centro de Ciências da Saúde, Fortaleza, CE - Brasil.

**Keywords:** Insuficiência Cardíaca/fisiopatologia, Volume Sistólico, Epidemiologia, Hospitalização, Mortalidade

O último levantamento epidemiológico da *American Heart Association* aponta que 6,2 milhões de americanos acima de 20 anos apresentam insuficiência cardíaca (IC), havendo projeção que esse número chegue a mais de 8 milhões em 2030.^1^ No Brasil, entre 2008 e 2017, a IC foi a principal causa cardiovascular de hospitalização, perfazendo 2,25% de todos os internamentos, com mortalidade de 14/100.000.^2^ Segundo Fernandes et al., essa taxa chega a 19,2/100.000 em estados menos desenvolvidos da federação.^2^

Diante da prevalência e gravidade da doença, o estudo “Características e Tendências na Mortalidade em Diferentes Fenótipos de Insuficiência Cardíaca na Atenção Primária” traz valiosos dados para melhor compreender, estratificar e tratar os pacientes com IC.^3^.

Descrita na última década, a insuficiência cardíaca com fração de ejeção intermediária (ICFEi) ocupou uma “zona cinzenta” de pacientes com fração de ejeção (FE) entre 41% e 49%, compreendendo aproximadamente 7% a 25% de todos os pacientes com IC. Trata-se de um grupo com características heterogêneas, que ora apresentam semelhanças com o grupo de pacientes com FE reduzida (ICFEr), ora com o grupo de FE preservada (ICFEp) e ora apresentam-se com um fenótipo único.^4^ Há quem advogue, inclusive, não se tratar de um grupo à parte, mas sim de um fenótipo de transição entre ICFEr e ICFEp.^5^

No estudo de Jorge et al.,^3^ a prevalência do fenótipo de ICFEi observada no serviço de atenção primária foi de 22%, próximo ao encontrado em outro estudo nacional, de Cavalcanti et al.,^6^ em que 26% dos pacientes com IC agudizada apresentavam o fenótipo intermediário.^6^ Tais frequências são superiores ao descrito por Peterson et al.,^7^ quando 17% dos pacientes atendidos por IC agudizada apresentavam ICFEi.^7^

É digno de nota citar que, dentro dessa nova categoria de IC, a literatura sugere subgrupos com diferentes prognósticos a partir da análise do comportamento dinâmico da FE: ICFEi deteriorada, ICFEi recuperada e ICFEi inalterada.^8^ Em trabalho de Savarese et al.,^9^ foram estudados 4.942 pacientes a partir do registro populacional *Swedish Heart Failure Registry* que apresentavam pelo menos dois ecocardiogramas consecutivos, realizados com intervalo médio de 1,4 anos. Foi analisada a incidência de transição entre grupos fenotípicos a partir de um aumento, queda ou manutenção da FE, bem como a implicação prognóstica dessas alterações. Os autores observaram os seguintes resultados: entre os pacientes com ICFEp, 21% deterioraram para ICFEi e 18% para ICFEr; entre aqueles com ICFEi, 37% deterioraram para ICFEr e 25% melhoraram para ICFEp; já entre pacientes ICFEr, 16% evoluíram para ICFEi e 10% modificaram para o grupo ICFEp. Pacientes que melhoraram a partir da ICFEr, passando a apresentar fenótipo de ICFEi ou ICFEp tiveram menor mortalidade e hospitalização, sendo o desfecho oposto para aqueles que apresentavam ICFEp ou ICFEi e passaram para o fenótipo ICFEr.^9^

A descrição da possibilidade dos três subgrupos acima talvez possa explicar as diferenças nos resultados entre os diversos estudos. Caso em uma dada pesquisa predominasse subgrupo que vinha com FE reduzida em recuperação, possivelmente eles teriam características mais similares às do grupo com FE preservada. De outra forma, caso predominasse subgrupo de ICFEi que originalmente apresentava FE melhor, porém evoluindo com gradativa piora, as características poderiam se assemelhar mais ao grupo com ICFEr. Importante ainda considerar que cálculos ecocardiográficos habituais da FE realizados nesses estudos apresentam limitações, têm resultados dinâmicos a depender das condições hemodinâmicas do paciente e apresentam variabilidade inter e intra-observadores. Para dirimir tais limitações, novas técnicas como o *strain* estão sendo incorporadas.^10^

O artigo que ensejou este editorial, pioneiro em território nacional no estudo da ICFEi, seguiu uma metodologia adequada e trouxe diversas informações que certamente auxiliarão em nossas abordagens clínicas. Entretanto, há que se analisar os dados no contexto onde a população estava inserida, ou seja, na atenção primária, o que pode diferir de uma análise global deste subgrupo.

Existem algumas limitações na interpretação dos resultados encontrados como: tamanho da coorte pequena diante da grande prevalência da doença, contando com apenas 51 diagnósticos em 560 pacientes; todas as avaliações clínicas, laboratoriais e ecocardiográficas foram feitas em uma única ocasião, não sendo possível avaliar a evolução desses parâmetros temporalmente; os dados dos fármacos em uso para IC também foram aferidos em uma ocasião, não sendo possível a análise se os resultados refletiram um tratamento médico otimizado, considerando a baixa taxa de uso dos principais fármacos na época da análise inicial do estudo; e a ausência de caracterização adequada dos graus de disfunção diastólica.

Há aspectos que chamam a atenção nas características dos grupos, sendo observado que metade dos pacientes com ICFEi usavam diuréticos, semelhante ao grupo com ICFEp e maior do que o grupo com ICFEr. Outro dado foi em relação à dosagem de BNP, que se mostrou menor na ICFEi do que no grupo com ICFEp. Esses achados conflitantes poderiam refletir no desfecho combinado que envolveu internações hospitalares, reduzindo a diferença entre os grupos.

A baixa taxa de uso de betabloqueadores no grupo com ICFEr, com apenas 36% e 30% nos demais grupos, mostra-se bastante preocupante, além da taxa de 60% a 70% do uso de IECA/BRA. O tratamento padrão para IC não estaria sendo realizado nas unidades de atendimento primário? Ou os grupos passaram a ter conhecimento das patologias ao serem introduzidos no estudo, sendo esses percentuais reflexo de uma análise inicial? Ambas as situações ensejam discussões sobre a necessidade da busca ativa desses pacientes e implementação de medidas efetivas para garantir o pleno uso das terapêuticas padrões no tratamento da IC.

Diante desses comentários, os achados do estudo precisam ser confirmados a nível nacional em análises maiores, em grupos não só de atenção primária, para que possamos entender como os nossos pacientes realmente estão sendo conduzidos, se dentro das evidências científicas maiores, principalmente em relação aos grupos de maior gravidade.

Portanto, parabenizo ao grupo pela iniciativa em trazer informações relevantes na triagem dos fenótipos de IC na atenção primária, no contexto de uma entidade clínica tão incidente, prevalente e com alta taxa de morbimortalidade, mesmo considerando-se os diversos recursos terapêuticos conhecidos.^11^
