# Supplementary material for: Insuficiência Cardíaca com Fração de Ejeção Intermediária - Condição Temporária ou um Grupo Específico?
Source: Arq Bras Cardiol. 2021 Aug 9;117(2):307–8. [Article in Portuguese] doi: 10.36660/abc.20210482 (PMC8395807; doi:10.36660/abc.20210482)
Supplement: Translation

# Translation: Heart Failure with Mid-Range Ejection Fraction – A Temporary Condition or a Specific Group?

**Affiliations:** 1 FortalezaCE Brazil Faculdade de Medicina da Universidade Federal do Ceará, Fortaleza, CE - Brazil.; 2 FortalezaCE Brazil Centro de Arritmia do Ceará, Fortaleza, CE - Brazil.; 3 FortalezaCE Brazil Hospital de Messejana Dr. Carlos Alberto Studart Gomes, Fortaleza, CE - Brazil.; 4 FortalezaCE Brazil Universidade de Fortaleza Centro de Ciências da Saúde, Fortaleza, CE - Brazil.

**Keywords:** Heart Failure/physiopathology, Stroke Volume, Epidemiology, Hospitalization, Mortality

The latest epidemiological report from the American Heart Association indicates that 6.2 million Americans over 20 years of age had heart failure (HF), with the projection that this number could reach 8 million by 2030.^1^ In Brazil, between 2008 and 2017, HF was the leading cardiovascular cause of hospitalization, accounting for 2.25% of all hospitalizations, with mortality of 14/100,000.^2^ According to Fernandes et al., this rate reaches 19.2/100,000 in less developed states of Brazil.^2^

Faced with the prevalence of the severity of this disease, the study “Characteristics and temporal trends in the mortality of different heart failure phenotypes in primary care” has brought valuable data to better understand, stratify, and treat patients with HF.^3^.

As described in the last decade, heart failure with mid-range ejection fraction (HFmrEF) has occupied a “grey zone,” in patients with ejection fraction (EF) between 41% and 49%, comprising approximately 7% to 25% of all patients with HF. It is a group with heterogeneous characteristics. At times, it shows similarities with the group of patients with HF with reduced EF (HFrEF); at other times, with the group with HF with preserved EF (HFpEF), and sometimes it presents as a unique phenotype.^4^ Some authors even argue that it is not a separate group, but rather a transition phenotype between HFrEF and HFpEF.^5^

In the study by Jorge et al.^3^, the prevalence of the HFmrEF phenotype observed in a primary care service was 22%, close to that found in another Brazilian study, by Cavalcanti et al.,^6^ where 26% of patients with acute HF presented the mid-range phenotype.^6^ These frequencies are higher than those described in the study by Peterson et al.,^7^ where 17% of patients treated for acute HF had HFmrEF.^7^

It is worth mentioning that, within this new category of HF, the literature suggests subgroups with different prognoses based on the analysis of the dynamic behavior of EF, as follows: impaired HFmrEF, recovered HFmrEF, and unchanged HFmrEF.^8^ The study by Savarese et al.^9^ evaluated 4,942 patients from the Swedish Heart Failure Registry who had at least 2 consecutive echocardiogram measurements with an average interval of 1.4 years. They analyzed the incidence of transition between phenotypic groups as increased EF, decreased EF, or stable EF, in addition to the prognostic implications of these changes. The authors observed the following results: of patients with HFpEF, 21% transitioned to HFmrEF, and 18% transitioned to HFrEF; of those with HFmrEF, 37% transitioned to HFrEF, and 25% transitioned to HFpEF; of patients with HFrEF, 16% transitioned to HFmrEF, and 10% transitioned to HFpEF. Patients who improved from HFrEF, transitioning to the HFmrEF or HFpEF phenotype had less mortality and hospitalization, and the outcome was the opposite for patients with HFpEF or HFmrEF who transitioned to the HFrEF phenotype.^9^

The description of the possibility of these 3 subgroups may explain the differences in results between different studies. If, in a given study, a subgroup with reduced EF in recovery predominated, they could possibly have characteristics that were more similar to those of the group with preserved EF. In another case, if there was a predominance of the HFmrEF subgroup that originally had better EF, but evolved with a gradual worsening, the characteristics could be more similar to the HFrEF group. It is also important to consider that the usual echocardiographic calculations of EF carried out in these studies have limitations and dynamic results that depend on the patients’ hemodynamic conditions, and they have inter- and intra-observer variability. To resolve these limitations, new techniques such as strain are being incorporated.^10^

The article that gave rise to this editorial, a pioneer in the study of HFmrEF in Brazil, followed adequate methodology, and it brought diverse pieces of information that will certainly assist our clinical approaches. However, it is necessary to analyze the data in the context where the population was inserted, namely, in primary care, which may differ from global analysis of this subgroup.

There are some limitations to the interpretation of the results found, including the following: small cohort size, given the high prevalence of the disease, with only 51 diagnoses in 560 patients; all clinical, laboratory, and echocardiographic evaluations were performed at a single moment, and it was not possible to assess the evolution of these parameters over time; data on use of medication for HF were also collection at a single moment, and it was not possible to analyze whether the results reflected optimized medical treatment, considering the low rate of use of the main drugs at the time of the initial analysis of the study; and the lack of adequate characterization of the degrees of diastolic dysfunction.

There are some aspects that warrant attention in the characteristics of the groups. It was observed that half of the patients with HFmrEF were on diuretics; this was similar to the group with HFpEF and higher than the group with HFrEF. Another piece of data was in relation to the dosage of BNP, which was lower in the HFmrEF group than in the HFpEF group. These conflicting findings could influence the combined outcome that involved hospital admissions, reducing the difference between groups.

The low rate of use of beta-blockers in the group with HFrEF, with only 36% and 30% in the other groups, is rather concerning, in addition to the 60% to 70% rate of ACEi/ARB use. Is standard treatment for HF not being fulfilled in primary care units? Or did the groups become aware of the pathologies upon being included in the study, with these percentages reflecting an initial analysis? Both situations give rise to discussions regarding the need to actively search for these patients and to implement effective measures in order to guarantee the full application of standard therapies in the treatment of HF.

In light of these observations, the findings of this study need to be confirmed on the national level in larger analyses, not only in primary care groups, so that we may understand how our patients are really being managed, whether in accordance with broader scientific evidence, especially in relation to groups with greater severity.

Therefore, I congratulate the research group for their initiative in bringing information relevant to the screening of HF phenotypes in primary care, within the context of this clinical entity which is so incident and prevalent and which has such a high morbidity and mortality rate, even considering the many known therapeutic resources.^11^
